# Current and Novel Approaches in Influenza Management

**DOI:** 10.3390/vaccines7020053

**Published:** 2019-06-18

**Authors:** Erasmus Kotey, Deimante Lukosaityte, Osbourne Quaye, William Ampofo, Gordon Awandare, Munir Iqbal

**Affiliations:** 1 West African Centre for Cell Biology of Infectious Pathogens (WACCBIP), University of Ghana, Legon, Accra P.O. Box LG 54, Ghana; kotey6croesuss@gmail.com (E.K.); oquaye@ug.edu.gh (O.Q.); gawandare@hotmail.com (G.A.); 2 Department of Biochemistry, Cell & Molecular Biology, University of Ghana, Legon, Accra P.O. Box LG 54, Ghana; 3 Noguchi Memorial Institute for Medical Research, University of Ghana, Legon, Accra P.O. Box LG 581, Ghana; WAmpofo@noguchi.ug.edu.gh; 4 The Pirbright Institute, Ash Road, Pirbright, Woking, Surrey GU24 0NF, UK; deimante.lukosaityte@pirbright.ac.uk; 5 The University of Edinburgh, Edinburgh, Scotland EH25 9RG, UK

**Keywords:** Influenza virus, vaccines, passive immunization, immunotherapeutics

## Abstract

Influenza is a disease that poses a significant health burden worldwide. Vaccination is the best way to prevent influenza virus infections. However, conventional vaccines are only effective for a short period of time due to the propensity of influenza viruses to undergo antigenic drift and antigenic shift. The efficacy of these vaccines is uncertain from year-to-year due to potential mismatch between the circulating viruses and vaccine strains, and mutations arising due to egg adaptation. Subsequently, the inability to store these vaccines long-term and vaccine shortages are challenges that need to be overcome. Conventional vaccines also have variable efficacies for certain populations, including the young, old, and immunocompromised. This warrants for diverse efficacious vaccine developmental approaches, involving both active and passive immunization. As opposed to active immunization platforms (requiring the use of whole or portions of pathogens as vaccines), the rapidly developing passive immunization involves administration of either pathogen-specific or broadly acting antibodies against a kind or class of pathogens as a treatment to corresponding acute infection. Several antibodies with broadly acting capacities have been discovered that may serve as means to suppress influenza viral infection and allow the process of natural immunity to engage opsonized pathogens whilst boosting immune system by antibody-dependent mechanisms that bridge the innate and adaptive arms. By that; passive immunotherapeutics approach assumes a robust tool that could aid control of influenza viruses. In this review, we comment on some improvements in influenza management and promising vaccine development platforms with an emphasis on the protective capacity of passive immunotherapeutics especially when coupled with the use of antivirals in the management of influenza infection.

## 1. Introduction

Influenza viruses are highly contagious pathogens that are associated with a year-round global record reaching nearly a million morbidities and half-a-million mortalities. Four types of influenza viruses (i.e., A, B, C, and D) have been identified. Influenza viruses C (isolated in pigs and humans) and D (isolated from cattle) are less common; typically, influenza virus C is associated with less severe illness [1,2]. On the other hand, influenza viruses A (infecting avian and mammals including human) and B (almost exclusively infecting humans and seals) account for the annual global burden of influenza [3,4]. The persistence of influenza viruses A and B has been attributed to their ability to evolve rapidly. Antigenic variabilities are also common with influenza viruses A and B, and these are partly as a result of a phenomenon called the antigenic drift, referring to amino acid changes that allows viral escape from neutralizing antibodies [5,6]. Such immune-escape mutants often tend to have a higher host-cell avidity (compared to the wild-type virus) in exposed or vaccinated host and vice-versa, in naïve host [7]. Studies by Fergusson et al. revealed that antigenic drifts in seasonal influenza viruses (H3, H1, and B) were estimated at fixation rates of 0.0037, 0.0018, and 0.0013 nucleotide substitutions per site per year (±0.001) respectively [8]. This supports the idea that antigenic drifts occur more frequently in influenza A viruses than influenza B viruses. In addition, high mutation rates cause a tremendous impact in efficacy of the seasonal influenza vaccines which comprise forecasted strains [9]. For instance, gain or loss of N-linked glycosylation sites in the hemagglutinin (HA) can also participate in the antigenic drift: Skehel et al. showed that a single D63N substitution in HA1 created a novel N-glycosylation site that allowed an antigenic variant of an H3N2 to escape neutralization by a monoclonal antibody [10]. In the same study, the authors further observed that the 1968 influenza epidemic strain (A/VIC/3/75) that had N63 (known glycosylation site), was also recognized (when un-glycosylated) by antibodies raised against viruses of two earlier epidemics. As illustrated, altering glycosylation patterns is one of the means used by viruses that results in potential cause of vaccine failure. 

Antigenic shift also allows influenza viruses to escape pre-existing immunity [11]. This mechanism is reliant on the ability of the eight genomic fragments of influenza viruses to reassort with genomes of other influenza viral subtypes. It occurs when two or more of these distinct viruses infect a common host and generate novel viral subtypes or strains [11,12]. Thus, antigenic shifts (principally underlying influenza A virus pandemics) and antigenic drifts (underlying vaccine mismatches against seasonal influenza A and B viruses) and a wide host-range (for influenza A viruses) all contribute to the recurring cases of influenza all year round [13,14]. Furthermore, antigenic drifts and shifts are particularly reasons for why there is an immediate need for highly efficacious intervention. We review here vital influenza management strategies, novel vaccine and antiviral development approaches with deliberation on those with prospects.

## 2. Current Influenza Vaccines

Three types of vaccines against influenza are currently used worldwide including inactivated influenza vaccine (IIV), live-attenuated influenza vaccine (LAIV) and influenza virus subunit vaccine: each of which has its own advantages and drawbacks. IIV is formulated with replication-incompetent virus, due to whole pathogen inactivation usually achieved by formaldehyde treatment or split virion vaccines generated by disruption of the viral membrane [15]. Intramuscular administration of the IIV has been shown to induce both local and systemic immunity [16]. However, to maintain the antibody titers, booster vaccinations are required. Additional considerations on the vaccine efficacy were raised following metadata analysis suggesting only 40% of children were being protected against influenza, with the percentages going a bit higher up to 65% for the adults [17,18]. LAIV comprise of re-assortant viruses generated from cold-adapted donor viruses (that contribute their internal genes) and identified virulent circulating strains of viruses (that contribute their HA and neuraminidase (NA)) as recommended by the WHO. Cold-adapted donor viruses are raised by several passages in embryonated chicken eggs with gradual reduction in temperature during every round of passage. By this process, re-assortant viruses that comprise of the LAIV can grow at 32–33 °C, the temperature range of cells lining the mucosal surfaces of the nasopharynx, when administered intranasally [19]. Replication of LAIV viruses in the nasopharynx elicit immune response that epitomizes a natural influenza infection. For this reason, LAIV has shown some superiority over the IIV in terms of the induction of mucosal immunity via secreted immunoglobulin A [19]. Use of the LAIV has proven to be safe in children (15 to 71 months) and immunocompromised persons (HIV-infected, chronic bronchitis and cystic fibrosis) [20,21,22]. The most spelt-out advantage is the “non-invasive” capacity of the attenuated viruses and this had made it suitable to use for all categories of vaccines, although LAIVs are not recommended for people with underlying chronic medical conditions [23]. A typical setback to the use of the LAIV is the possibility of the attenuated virus undergoing some genetic modifications and consequently reverting to virulence, a case which has not been reported for the LAIV [7]. Furthermore, since vaccine viruses are grown in eggs, there have been several concerns about allergic reactions among certain vaccines: whereas the LAIV ovalbumin contents (responsible for the vaccine allergies) are variable, other studies have showed that, the IIV contains a tolerable ovalbumin content of about 0.7 μg/ mL [24,25,26]. The development of the subunit influenza vaccines, which often comprise of influenza virus HA that have been purified following protein expression in cells, could be a means to avoid adverse reactions in people with egg allergies [27]. Besides, the subunit vaccine also offers desirable protection against seasonal influenza viruses; but its downside being higher dosage requirement at multiple times for full potentiation of immune protection comparable to that elicited by whole-virus vaccines [28].

It is worth noting that both the LAIV and IIV are cocktails of circulating seasonal influenza viruses. Mainly three viruses, i.e., A (H1N1) pdm09, A (H3N2), and the pre-determined dominant influenza B lineage (whether Yamagata or Victoria) are the constituents of the seasonal trivalent influenza vaccines (TIV). Subunit vaccines are also formulated as TIV, containing the HA of all representative vaccine strains [29]. However, it became necessary to feature both lineages of influenza B viruses based on the current global epidemiology of influenza as recommended by Ambrose and Levin in 2012. This resulted in the advancement of quadrivalent influenza vaccines (QIV) containing the pre-determined representatives of both Yamagata and Victoria influenza B virus lineages, in addition to the two pre-determined circulating seasonal influenza A subtypes [30]. This approach of vaccine preparation thus requires a constant reformulation to maintain desirable efficacy limits during an influenza season [30,31]. 

## 3. Use of the Seasonal Influenza Vaccines

Due to continued burden of infection with influenza, the US CDC advocates the use of seasonal influenza vaccines in all persons >6 months prior to the winter [32]. On the other hand, the WHO extends recommendations for the use of influenza vaccination in the persons categorized as high-risk, which comprises children >6 months, persons with chronic diseases, pregnant women, and healthcare and nursing workers [33]. However, in some parts of the world, mostly Africa and Asia, there are either limited or no established influenza vaccination policies. Thus, restricted availability of influenza vaccines makes vaccinations quite uncommon to these populations. Perhaps, such vaccination policies might not have been considered due to the cost of acquiring vaccines annually or still, the reduced efficacy of the influenza vaccines, as have been critically assessed by Xu et al., where recommendations have been made for twice-dose vaccination due to frequencies of seasonal influenza occurrences, all year round [34]. Although poor vaccine coverage in African countries was previously reported by Duque et al., upon investigation on the availability of seasonal influenza vaccines, there are still no clearly underpinned core reasons [35]. Therefore, the improved efficacy of influenza vaccines would also contribute to enhanced vaccine coverage in Africa and Asia.

## 4. Novel Influenza Vaccine Platforms

Rapid influenza virus evolution and yearly vaccine reformulations make the stockpiling of vaccines for future use a complicated issue. This subsequently delays preparation against any unforeseen epidemics. Therefore, lots of research now focuses on the development of novel broadly protective vaccine platforms, with hopes of enhancing both immunogen delivery and consequent immune response to select antigens. Some of these platforms include virus-like particle vaccines (VLP), synthetic virus vaccines, epitope vaccines, antigen-presenting cell inducible vaccines, COBRA vaccines, nanoparticle-based vaccines, and viral-vectored vaccines (Table 1).

## 5. Virus-Like Particle (VLP) Vaccines 

Virus-like particles are non-infectious multimers of viral surface glycoproteins that have the propensity to self-assemble [36]. VLPs are designed to maintain their native viral structure, but without their complete set of genetic materials. A typical influenza VLP has the HA, NA, and the matrix protein 1 (M1). Typically, plasmid constructs of the HA, NA, and M1 are used to transfect cells: resulting in formation of the capsid displaying surface proteins HA and NA [37]. VLPs have been proposed to be efficient vaccines against a range of viruses including human papilloma virus (HPV) and hepatitis B virus (HBV) or hepatitis E virus (HEV) as discussed by Zhao et al. [38]. Although these examples have completely different “biologies” when compared with the influenza virus, rapid advances and need for a new vaccine platform are generating some promising data for influenza VLPs. For instance, to overcome challenges raised by rapid influenza evolution Gao et al. attempted to generate VLPs with HBV backbone containing matrix protein 2 ectodomain (M2e) together with the epitope of highly conserved nucleoprotein (NP) [39]. Mice immunization with chimeric VLPs induced humoral as well as cell-mediated immunity and resulted in cross protection against several strains of virus [39]. Another approach for generation of VLPs is via combination of distinct HAs. Such technique has been described by Kapczynski et al. who upon co-expression of three different clade H5 HAs, a single NA protein and retroviral gag protein managed to generate triple-clade VLPs that were shown to protect chickens against lethal challenge [40]. For heightened immunity (involving both innate and adaptive), VLPs may be adjuvanted with various Toll like receptor (TLR) ligands, as demonstrated with the modified salmonella flagellin acting as a TLR5 ligand described by Wang et al. which resulted in highly specific immunoglobulin response [41]. Also, a GPI-anchored CCL-28 that was incorporated into the VLPs boosted IgA secreting cell migration, which increased murine mucosal immunity to both drifted and homologous influenza A (H3N2) viruses, as well as the longevity of protection [42]. VLPs thus provide a platform for improved formulation of multivalent (containing heterologous epitopes) influenza vaccine. 

## 6. Computationally Optimized Broadly Reactive Antigen Vaccines (COBRA) Vaccines

Computationally optimized broadly reactive antigen vaccines (COBRA) comprise VLPs that carry a computationally designed HA. Ted Ross’ group first generated consensus amino acid HA sequences of clade 2 highly pathogenic A (H5) involved in human infections and formulated VLPs to express this HA [43]. H5 COBRA VLPs potently induced HA-neutralizing antibodies, which provided efficient protection of both immunized mice and ferrets in a pathogenic H5N1 challenge experiment [43]. A similar approach also demonstrated protection of cynomolgus macaques [44]. The ability of the COBRA VLPs have since been demonstrated as a powerful system that induces a strong broadly neutralizing antibody response against multiple clades of H5N1 of viruses and multiple isolates of H1N1 viruses [45,46]. Similarly developed H1N1 and H3N2 COBRA vaccines have also been shown to induce broadly neutralizing antibodies in either mice or ferrets, against a broad spectrum of H1N1 and H3N2 viruses, respectively [46,47].

## 7. Synthetic Influenza Virus Vaccines

Several approaches have been tried in order to generate attenuated viruses using reverse genetics technology. A suggested technique to downregulate viral protein synthesis is via biased virus codon sequences. Average codon frequency alteration can result in attenuation of virus in mice models, as shown by Fan et al., suggestive that the avian codon–biased vaccine candidate that was fitter in eggs, is good news for generation of influenza vaccines in eggs [48]. Alternatively, replication-incompetent viruses can be generated upon truncation or knockdown of non-structural viral protein 1 (NS1)―a notable inhibitor of the host-protective interferon-induced immunity [18]. This has led to phase I/II clinical trial of a trivalent vaccine which revealed protection of vaccinees against the seasonal influenza viruses [49,50]. This platform has also paved way for the generation of single-cycle replicating influenza viruses as vaccine candidates and have shown similar or higher protection than the conventional LAIV [51,52,53]. The main reasons synthetic influenza vaccines remain promising is due to their ability to alter viral immunomodulatory traits and their amenability to rapid production of vaccines.

## 8. Epitope Vaccines

Epitope-based vaccines can serve both immune refocusing role as well as targeting integral virus specific epitopes. Remarkable work inspired by the stabilization of respiratory syncytial virus fusion protein (F) has shown that the relatively conserved HA stem could induce broadly neutralizing antibodies that are protective in mice and non-human primates against several virus subtypes harboring group I HAs (H1, H2, H5, and H9) and group 2 HAs (H3 and H7) [54,55,56]. In order to characterize an epitope of limited variability that are located on the HA head, Thompson et al observed that sera collected from young children during a pandemic revealed a cross-reactivity pattern to historical influenza A (H1N1) isolates [57]. A conserved epitope situated on the HA head was confirmed further, demonstrating the protective capacity of this epitope in a murine challenge against diverse strains of the influenza A (H1N1) [57]. Another universal vaccine candidate currently undergoing phase III clinical trials is based on *Escherichia coli*-expressed artificial recombinant protein consisting the concatenation of nine linear epitopes (five of which are specific to HA; three, to NP and one, to M1) of several influenza virus strains, and this vaccine has been shown to induce both cellular and humoral immunity in mice and it is envisaged as able to overcome high virus mutation rates [58]. The epitope-based vaccination therefore affords the direct involvement of B and T lymphocytes that are both required for effectual control of viruses during an infection. 

## 9. Antigen-Presenting Cell (APC) Inducible Vaccines

Recently, more focus is drawn to increasing the abilities of antigen-presenting cells (APCs) to efficiently involve the T-cell arm of immunity to influenza virus clearance. One of the examples is the work of Fonteneau and colleagues who showed that after exposure to influenza virus, dendritic cells (DCs) (both CD11c^+^ DCs and plasmacytoid DCs) induced an expansion of anti-influenza virus cytotoxic T lymphocytes (CTLs) and T helper 1 (TH1) CD4^+^ T cells [59]. Inspired by the previous findings, Abdel-Motal et al. also demonstrated that grafting the alpha-Gal epitope onto HA promoted its opsonization thereby enhancing the uptake of the vaccine virus by APCs [60]. Importantly, work by Grødelang et al. showed that it is possible to target the HA to different surface molecules on antigen presenting cells and thereby orient the immune response towards either an antibody/Th2 response or a CD8^+^/Th1 T cell response [61]. There is a variety of approaches to target APCs including antibody, nanoparticle, or ligand-mediated methods that emphasize APC inducible vaccine universality.

## 10. Nanoparticle-Based Influenza Vaccines

Continued efforts to develop a universal influenza vaccine has driven the use of self-assembling monomeric ion-carrier molecules, called Ferritin for administration of multivalent vaccine constructs. In vivo assessment of nanoparticle-based vaccines displaying multivalent HA from 8 diverse strains of H1N1 influenza A viruses, were shown to induce broadly protective antibodies in mice, whose protection spanned strains from 1918 through 2009. The breadth of protection by the nanoparticle-induced antibodies were also shown to be more profound in comparison to the individual components of the conventional multivalent vaccine [62]. Tao and Gill also immobilized the matrix protein 2 extracellular domain (M2e) that resulted in increased induction of M2e-specific antibodies affording protection of mice challenged with virulent strain of an influenza virus [63,64]. Intranasal administration of polylactic-co-glycolic acid (PLGA) nanoparticle conjugated to influenza A (H1N1) conserved peptides as a vaccine were also shown to induce protection in the lungs of pigs, via the induction of antigen-specific CD4^+^ and CD8^+^ T cells [65]. A similar approach by Chahal et al. also demonstrated the induction of both CD8^+^ and antibody responses in mice; this was separately challenged with either viruses (i.e., H1N1 and Ebola) or a parasite (*Toxoplasma gondii*) after immunization with nanoparticle formulation that involved a single or combination of gene-specific RNAs encapsulated in a dendrimer [66]. Recently, a double-layered protein nanoparticle developed using tandem expressed M2e (comprising human, avian, swine, and domestic fowl), with or without recombinant HA stalk proteins from H1 and H3, showed homosubtypic and heterosubtypic protection in mice that were immunized prior to challenge with specific influenza A viruses [67]. Though a promising influenza vaccine platform, high-throughput nanoparticle-based vaccines approaches that will facilitate replacement of the seasonal influenza vaccines are still to be developed [68].

## 11. Viral-Vectored Vaccines

Viral-vectored vaccines platforms are designed to mimic natural infections, in that viral molecules are displayed on either similar or dissimilar virus. This approach has been shown to involve both the humoral and cellular immunity [69]. Use of the modified vaccinia virus Ankara (MVA) as a vector, incorporating influenza NP and M1 proteins, has shown potent induction of both influenza virus-specific humoral and cell-mediated responses leading to the phase II clinical trial [70,71]. An Adenovirus vectored influenza vaccine has equally shown a strong induction of influenza virus HA-stalk cross-reactive antibodies in mice [72]. Recently, Lingel et al. expressed multivalent adenovirus vectored influenza virus vaccine (that comprise the consensus sequences of divergent H1, H2, H3, and H5 HAs) and demonstrated that low dose administration in mice conferred protection of mice in a lethal influenza virus challenge experiment [73]. This provides an avenue for scaling up appropriate doses for later use in seasonal vaccine development efforts [73]. The alphavirus, baculovirus, Newcastle disease virus, pox virus, parainfluenza virus and vesicular stomatitis virus, are other vectors that have also been proposed to be applicable in vectored vaccines [74].

## 12. Current Influenza Managing Antivirals

Management of ongoing influenza infection currently requires the use of antiviral drugs. Two drug classes approved for the control of influenza infections include the adamantanes (Amantadines and Rimantadines) and NA inhibitors (Oseltamivir, Zanamivir, Laninamivir, and Peramivir). Whereas Adamantanes target the ion channel M2, which is involved in the release of viral ribonucleoprotein complexes in the host cell, NA inhibitors act by competitively engaging viral NA protein that is otherwise responsible for newly generated virion dissemination [75,76,77,78]. However, influenza viruses resistant to both the adamantanes and neuraminidase inhibitors have emerged rapidly [79,80,81,82,83,84,85,86]. This shows need for search of either novel antivirals or other viral or host targets, which can be used for the development of next-generation drugs [87]. Additionally, lack of data suggesting antiviral efficacy against highly pathogenic avian influenza viruses (HPAI), i.e., H5N1 remains an important issue in areas with possible spillover events from avian into humans [88]. It is imperative to note that there are still NA inhibitor-sensitive influenza A viruses in circulation, and this drug class can be extremely helpful in the management of an outbreak or even, a pandemic in the absence of highly efficacious vaccines. 

## 13. Novel Influenza Management Therapies

### 13.1. Next-Generation Antivirals Against Influenza

The burden of influenza requires identification of a novel compounds that have the potential to alleviate the symptoms and reduce viral shedding. New research focuses are developing not only virus targeting antivirals but also the ones that target the host organism (Table 2). For instance, DAS-181-F03/F04 is a recombinant host-sialic acid targeting molecule acting as a sialidase at the surface of host’s susceptible cells such as the epithelial cells of the airways [89]. This inhibits the initial attachment of the influenza virus HA that recognizes Neu5Ac in the α(2, 3)- and α(2, 6)-linked configurations of sialic acids [90]. The universality of DAS-181-F03/F04 is due to its ability to act on both types of sialic acids and, therefore, it can be used in avian―carrying α(2, 3), and mammalian―containing α(2, 6), hosts and was shown to inhibit H1N1pdm09, H3N2, and H5N1 viruses [91,92,93]. Besides influenza viruses, other sialic acid-dependent viruses such as human metapneumovirus and parainfluenza III virus were also shown to be inhibited by DAS-181-F03/F04 [94]. Another host targeting antiviral is the Nitazoxanide which falls under a category of thiazolides that are known to produce active metabolites following deacetylation. These metabolites have been shown to inhibit the maturation of influenza HA by blocking the trafficking and insertion of HA onto the host cell surface [95,96]. Nitazoxanide is a licensed anthelminthic drug that has been repurposed to ameliorate influenza due to its broad range of protection efficiency against influenza viruses in a phase II b/III clinical trial [95]. The antiviral, JNJ-63623872 (Pimodivir) is non-nucleoside influenza virus PB2 inhibitor, which binds a conserved domain on the polymerase subunit, PB2 of influenza A viruses and thereby inhibiting host cap-snatching (a perquisite for the initiation of viral replication). Pimodivir has been shown to be efficacious in nanomolar concentration during both prophylaxis and treatment or in co-administration with the neuraminidase inhibitor (oseltamivir) in mice models [97,98]. Phase II clinical trial has also confirmed the efficacy of Pimodivir when used as a single drug or when co-administered with Oseltamivir. In addition to this, drug effect was not associated with any detectable interference of any cellular processes [99,100]. There is currently an ongoing recruitment for phase III interventional trial of Pimodivir among adolescents, adults and the aged with non-hospitalized participants with chances of developing complications. Meanwhile, a pre-approval trial (of Pimodivir) for the treatment of patients with influenza virus A (H7N9) infection, has been allowed. 

In 2002, Furuta et al. discovered the anti-influenza virus drug T-705, during screening of anti-influenza compounds by plaque reduction assay. T-705 was shown to have a selective index over 2000 for influenza viruses, with no detectable cytotoxicity in vitro [101]. Trials of T-705 in mice confirmed both selectivity to influenza viruses and protection as an anti-influenza virus therapeutic agent. Along the same lines, Furuta and colleagues observed some inhibitory action of the drug to some other RNA viruses, but not in DNA viruses. The mechanism of action of the drug has been attributed to the inhibition of viral RNA-dependent RNA polymerase by the active phosphoribosylated T-705, which acts a nucleotide analogue and, hence terminating viral replication [102,103,104,105]. These have warranted further experiments on many other RNA viruses possessing either negative-strand segmented RNA genomes such as arena-and bunya-viruses or positive-strand RNA such as noro- and flavi-viruses [106,107,108,109]. In summary, T-705 is effective against influenza viruses in group 1 such as H1N1pdm09, H5N1 and group 2 such as H7N9 and also drug-resistant strains of these viruses and has been exploited for the treatment against other viruses e.g., Ebola virus [110], and to date, no known resistance has been reported, except for a purposeful mutation that conferred resistance to a laboratory H1N1pdm09 virus strain [111] Efficacy of T-705 for treatment of influenza has thus warranted its advancement through phase III and II trials in Japan and US respectively [112].

Baloxavir marboxil is another antiviral which was first developed in Japan and was shown to act as a selective cap-dependent endonuclease inhibitor of influenza viruses’ (both A and B) polymerase subunit PA. The drug had exhibited preferable safety, tolerability, and pharmacokinetic properties in a phase I trial [113]. The overall optimal performance against uncomplicated influenza among adults and adolescents, was shown during phase II and III trials that involved Japanese and Americans respectively [114]. The drug has currently been approved and marketed in the US as Xofluxa, for the treatment of acute uncomplicated influenza among ≥12 years [115].

Arbidol (Umifenovir) is another influenza-limiting drug that had previously been licensed for use in both China and Russia, for almost several decades [116,117]. It was originally developed in Russia and was found to potently inhibit influenza virus fusion with susceptible cell membranes, followed by interferon induction [118]. Like other broad-spectrum antivirals discussed earlier, besides influenza viruses, Arbidol has been shown to efficiently suppress other viral infections caused by paramyxoviruses and picornaviruses, bunya viruses, rhabdoviruses, reoviruses, togaviruses, hepaciviruses, Ebola virus, arenaviruses, herpesviruses, and the flaviviruses (Zika virus, West Nile virus and Tick-borne encephalitis virus) [119,120,121]. Currently, phase III trial of the Arbidol in ongoing in China and the drug is also due phase IV trial (with an unknown status) in Russia.

Ingavirin, a drug developed in Russia, adds on the current list of influenza-limiting antivirals due to its direct interference with the transportation of newly synthesized viral NP [122,123,124]. As of 2017, Ingavirin has been approved for the treatment of influenza and other viral causes of acute respiratory illness, in Russia, following the completion of phase IV trial. 

### 13.2. Passive Immunotherapeutics for Management of Influenza

**Figure 1:**
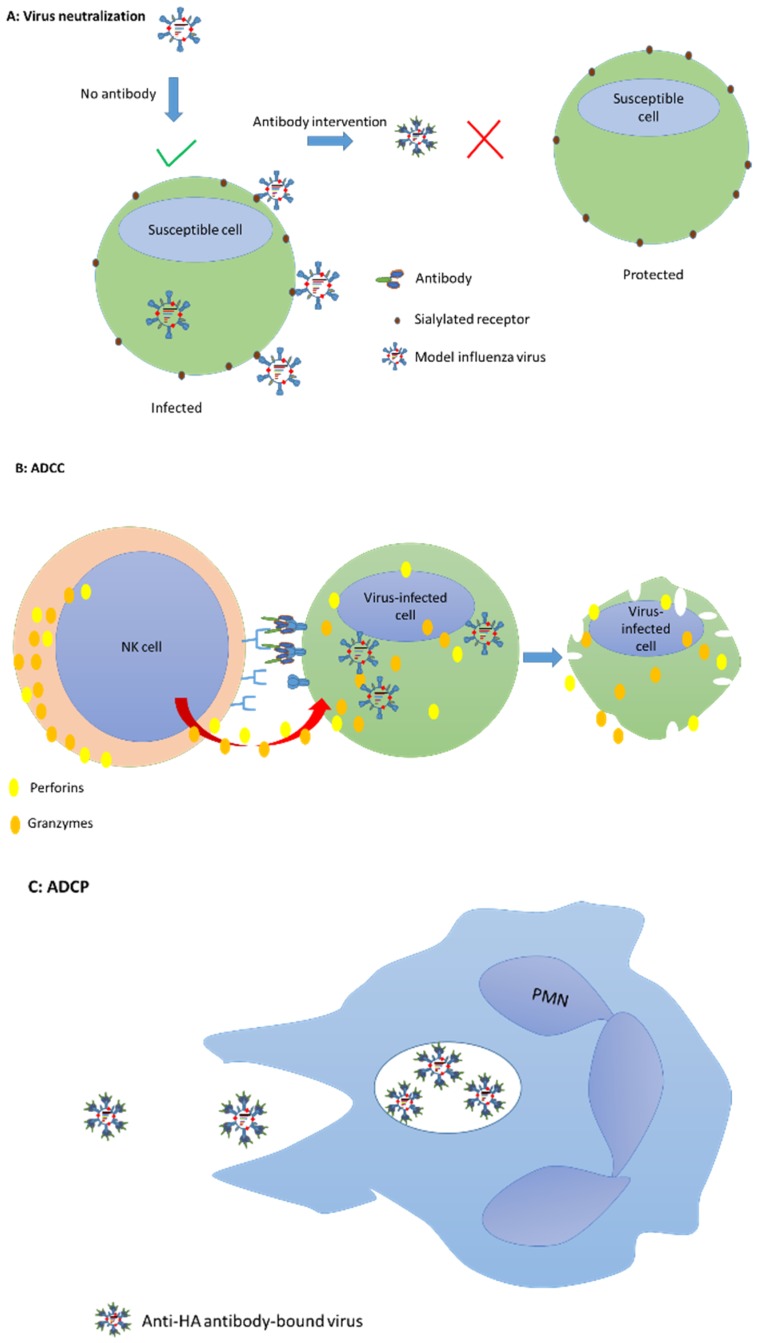

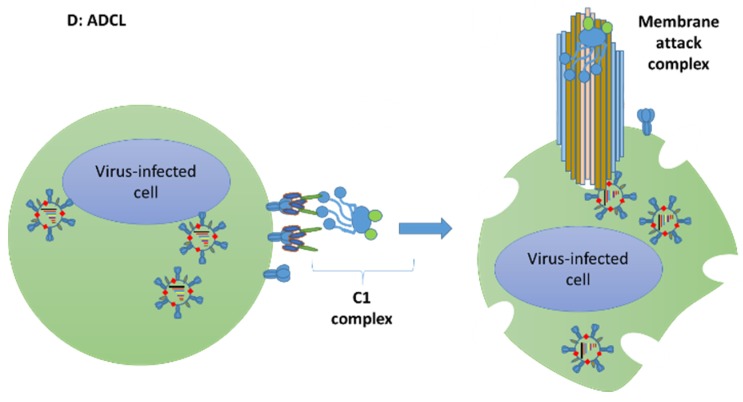
Mechanisms of antibody protection via passive immunization. This figure outlines the possible mechanisms by which antibodies could mediate instant protection when administered either as a prophylaxis or treatment. (**A**) Broadly neutralizing antibodies interact with HA interfering with the virus attachment to host cell. (**B**) Opsonized infected host cells attract natural killer (NK) cell destruction via the process of antibody-dependent cellular cytotoxicity (ADCC). (**C**) Opsonized virus particles activate their phagocytosis by polymorphonuclear cells (PMN) via the process of antibody-mediated cell phagocytosis. (**D**) Virus infected cells displaying the surface proteins of replicating viruses attract the assembly of the classical complement proteins forming a membrane attack complex that destroys the cell by osmosis in a process called antibody-dependent cell lysis (ADCL).

The need for new strategies to control influenza infections has led to the investigations of antibody therapy potential. Such an approach is based on neutralizing monoclonal antibody (mAB) expression and delivery into the host pre- or post- exposure to the pathogen (Figure 1). There are several clinical trials testing mAB efficacy against infectious pathogens, including the TNX-355 (Ibalizumab) which has been successfully approved for the use in HIV-infected patients and Palivizumab for the treatment of respiratory syncytial virus infections [125,126]. The feasibility of immunotherapy for rapidly evolving influenza was attained upon the discovery of broadly neutralizing antibody C179 isolated from a mouse immunized with H2N2 antigen [127]. Further characterization showed the binding of C179 to the stem of HA, thus providing a structural basis to its ability to inhibit fusion [127,128].

Techniques to fully recover human antibodies were further improved by Throsby et al. who used human memory B^+^ cells and phage panning to recover thirteen mABs one of which CR6261 entered clinical trials [129]. CR6261 as well as C179 was later found to neutralize only influenza A viruses with group 1 HA. Similarly, another potential mAB MEDI8852 inhibiting cleavage of HA0 was also isolated from human memory B^+^ cells and has completed phase IIa clinical trials [130,131]. This antibody can not only neutralize viruses of different phylogenetic groups but can also overcome amino acid changes due to its binding flexibility as shown by X-ray structures. Following on, the repertoire of broadly neutralizing antibodies was characterized, using plasma cells [132]. Discovery of mABs that can cross-neutralize multiple viral subtypes could lead to novel passive immunotherapy treatments for human infections and could also have the potential to abrogate spillover infection events from zoonotic species. Several example antibodies with the highest potential for use in management of influenza, are listed in Table 3.

Experiments by Lu and colleagues demonstrated that the Fragment antigen-binding, F(ab’) 2 region of an equine anti-H5N1 antibody could protect mice against a lethal challenge of influenza H5N1 [133]. Such an example shows that use of passive immunotherapy during influenza outbreaks could complement the use of available antivirals that would increase the survival in both humans and other animals. 

Most antibodies tend to be elicited against the more antigenically variable head domain of the major surface glycoprotein HA. On the other hand, the less variable stalk domain is characterized as sub-immunodominance and associated with minimal immune response [134,135]. Experiments by Margine and colleagues employing the variable heads but constant H3 stalk domain as a vaccine in mice, triggered broadly cross-reacting stalk-based antibodies [136]. Wohlbold et al. also realized the importance of the influenza HA stalk as a good vaccine target when they ascertained the transmission-blocking capacity of stalk-directed vaccines in ferrets [137,138]. In addition, studies by El Bakkouri et al. used an immune serum of mice (previously immunized with three tandem copies of M2e, fused with the hepatitis B virus (HBV) core fusion protein) to show Fc-dependent immunity against the M2e [139]. Intranasal administration of monoclonal anti-NA antibody resulted in total protection (in mice) with significantly lower virus titers and no viral escapes as determined by deep sequencing of viral genomes [140].

Passive immunotherapy has demonstrated rapid relief and life-saving capabilities in the treatment of viral infections like measles, rabies, HBV and others [141,142,143]. As established for RSV with the commercial immunotherapy palivizumab, antibody-based therapies with influenza antibodies could aid most susceptible populations including infants, which often suffer from influenza-related complications. Considering this, prophylactic use of antibodies should be considered as a part of an immediate management and care procedures. One of the approaches considered for antibody delivery is via recombinant virus vectors. Balazs et al. developed adenoviral-vectored human broadly neutralizing antibody (F10 or CR6261) which conferred protection in mice (who received the construct intramuscularly) during a pathogenic influenza virus (H1, H2, and H5) challenge [144]. Their experiments also showed equally protective amounts of the intramuscularly expressed F10 antibodies in the sera of both young and old mice and non-obese diabetic/ severe combined immunodeficiency/interleukin receptor subunit gamma null mice, suggesting how efficacious passive immunotherapy could be for both the aged and immunodeficient [144]. More so, antibody-based immuno-prophylaxis and -therapeutics have more prospects in effective intermediation of influenza outbreaks and production of specific influenza vaccines, noting that the broadly-reacting antibodies have the capacity to both inhibit virus replication and shedding [145]. A more consolidating evidence of passive immunotherapeutic management of a case infected with A(H5N1) which was described to have encountered rapid relief in less than two days after convalescent plasma administration, and interestingly, resulted in no detectable viruses by real-time polymerase chain reaction [146]. Furthermore, it is worth noting the diverse protective mechanisms by which antibodies can exert their functions directly on pathogens or on pathogen-infected cells: virus neutralization, antibody-dependent cell-mediated cytotoxicity (ADCC), antibody-dependent cell phagocytosis (ADCP), and antibody-dependent cell lysis (ADCL) are well-studied in the context of influenza viruses. ADCC, which mainly involves destruction of infected cells chiefly by natural killer (NK) cell activity (via perforin and granzyme B secretion into infected cells leading to cell lysis with destruction of intracellular pathogen), via recognition of antibody Fc that cross-links NK cell Fc receptors (FCRs), has been observed for murine antibodies weakly interacting with cognate influenza M2e [147]. Similarly, virus replication in mice was shown to be suppressed due to neutrophil-antibody based enhanced phagocytosis (ADCP) in pulmonary infected mice that either received anti-influenza serum before or after influenza infection, when neutrophils were retained and not inhibited by antibodies [148]. In direct virus neutralization, antibodies that bind the receptor-binding site (or nearby site) of hemagglutinin prevent the viral attachment to the host cell: these antibodies, that are mainly targeted against the globular head of HA, are elicited by either vaccination or natural infection [145]. ADCL is another mechanism that could augment the killing of influenza viruses as observed by Terajima et al., realizing that mostly neutralizing human monoclonal antibodies (either recognizing the globular head or stalk of HA) exerted this kind of effect, contrary to previously associated stalk-specific antibodies only [149].

Above all, we emphasize the therapeutic capacity of novel monoclonal antibodies in combating influenza infection, and speculate that, in addition to the use of effective antivirals, passive immunotherapy against both influenza A and B viruses might be the way forward for influenza virus management amongst all classes (be it high- or low-risk) of patients. We envisage that the involvement of passive immunization will culminate in an accelerated relief through any of the mechanisms previously described, and this provides allowable time for the full activation of the adaptive immune system via the conventional antigen-presenting mechanisms. Additionally, infected persons who were passively immunized could develop a natural immunity to the specific viruses and this immunity could be long-lasting giving protection to other antigenically-matched strains [150]. Also, the association of passive immunization with rapid relief increases the chances of abating the evolution of escape mutants suggested to arise due to vaccination and its consequent herd immunity [151].

## 14. Conclusions

Improving vaccine delivery and efficaciousness is paramount to combat continuous circulation of epidemic influenza; however, in addition to the ongoing deployment of novel anti-viral strategies which include the highly promising immune therapies for treatment and prophylaxis are vital to limit and protect against the on-going threat of pandemic influenza. 

## Figures and Tables

**Table 1:** Summary of novel influenza virus vaccine platforms.

Vaccines	Design	References
Virus-like particle vaccines (VLP)	Self-assembling viral matrices that express a single or multivalent viral surface proteins	[36,37,38,39,40,41,42]
Computationally optimized broadly reactive antigen vaccines (COBRA)	VLPs bearing computationally optimized viral surface proteins	[43,44,45,46,47,48,49]
Synthetic virus	Generation of replication-incompetent viruses bearing genetically attenuated genomic sequences	[18,49,50,51,52,53,54]
Epitope	Epitope-rich proteins of viruses, designed to induce protective epitope targeted antibodies	[55,56,57,58,59]
antigen-presenting cell (APC) inducible	APC-targeted delivery of immunogenic viral proteins to induce quicker and T cell responses	[60,61,62]
Nanoparticle-based	Self-assembling nano-molecules that carry a single or multivalent viral surface protein	[63,64,65,66,67,68,69]
Viral-vectored	Mainly involves use of dissimilar viral matrices as carriers of specific viral protein	[70,71,72,73,74,75]

**Table 2:** Influenza antiviral drugs approved or in clinical trials.

Antiviral	Mechanism of Action	Clinical Phase and Status	Country of Development/Trial
Das181 (Fludase)	Sialic acid removal in the respiratory airways	II (IFV), III (PIV) not yet recruiting	USA
Nitazoxanide	HA maturation inhibition	III completed	USA
JNJ-63623872 (Pimodivir)	Small molecule inhibitor of influenza A virus PB2	III recruiting	Belgium
T705 (Favipiravir)	RNA-dependent RNA polymerase inhibitor	IV	Japan
Baloxavir marboxil	Small molecule inhibitor of cap-dependent endonuclease (PA)	III recruiting children <1 year ^1^	Japan
Arbidol (Umifenovir)	HA resistance to conformational changes triggered by pH	III recruiting in China/IV unknown status in Russia	China; Russia
Ingavirin	Interaction with NP and inhibition of viral genome release	IV completed	Russia

Note: Drugs and their clinical statuses were adapted from the clinicaltrials.gov. ^1^ Approved for treatment of acute uncomplicated influenza among ≥12 years

**Table 3:** Antibodies undergoing clinical trials.

Antibody	Target/Mechanism of Action	Clinical Phase Status	Country
MEDI8852	HA stem	IIa completed	USA
MHAA4549A	HA stem	II completed	USA
VIS410	HA stem	II completed	USA
Intravenous hyper-immune immunoglobulin (IVIG)	Antigen specific antibody pool with neutralizing potential	III recruiting	USA
Ergoferon	Suppression of non-specific immune activation (by any virus)	IV completed	Russia
CR6261	“highly conserved membrane-proximal stem of H1 and H5 viruses’ HA1 and HA2”	II completed	USA

Note: Information was retrieved from the clinicaltrials.gov.
